# Photoelectrochemical Detection of Calcium Ions Based on Hematite Nanorod Sensors

**DOI:** 10.1021/acsanm.2c03978

**Published:** 2022-11-10

**Authors:** Bo Zhou, Yunlu Jiang, Qian Guo, Anirban Das, Ana Belén Jorge Sobrido, Karin A. Hing, Anatoly V. Zayats, Steffi Krause

**Affiliations:** † School of Engineering and Materials Science, Queen Mary University of London, Mile End Road, London E1 4NS, U.K.; ‡ Department of Physics and London Centre for Nanotechnology, King’s College London, Strand, London WC2R 2LS, U.K.

**Keywords:** α-Fe_2_O_3_ (hematite) nanorods, photoelectrochemical sensing, calcium ion (Ca^2+^) sensing, light-addressable potentiometric sensor, light-activated electrochemistry

## Abstract

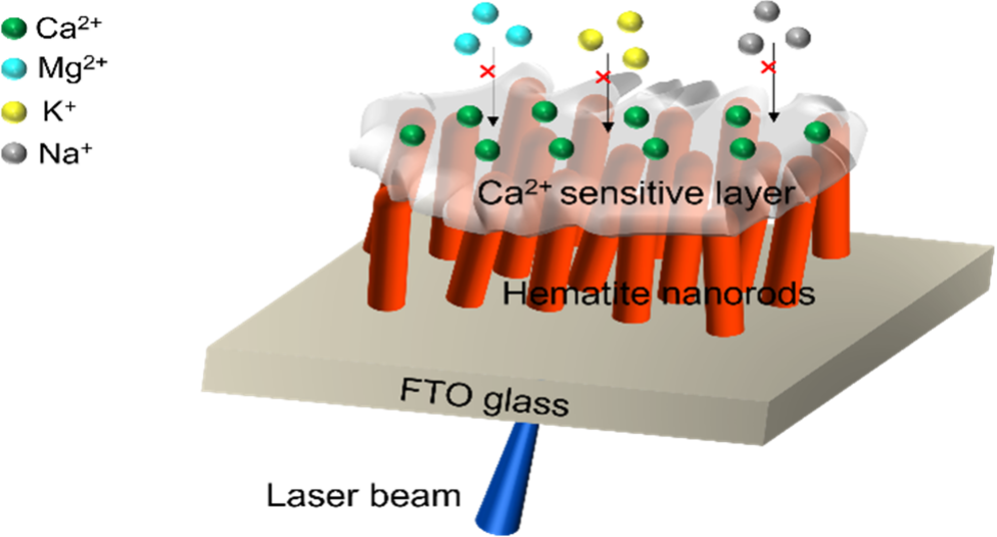

α-Fe_2_O_3_ (hematite) thin films have been shown to be a robust sensor substrate for photoelectrochemical imaging with good stability and high spatial resolution. Herein, one-dimensional (1D) hematite nanorods (NRs) synthesized via a simple hydrothermal method are proposed as a substrate which provides nanostructured surfaces with enhanced photocurrent responses compared to previously described hematite films, good stability, and excellent spatial resolution for potential imaging applications. The photoelectrochemical sensing capability of hematite NRs was demonstrated by a high pH sensitivity without modification. The modification of the hematite NRs with a thin poly(vinyl chloride) (PVC)-based ion-selective film allowed highly reversible amperometric detection of calcium ions with sensor materials traditionally employed in potentiometric devices.

## Introduction

Calcium ions (Ca^2+^) have a key role in both the intracellular and extracellular signaling of cells that affect every aspect of cellular life such as gene expression, protein secretion, cell adhesion, differentiation, proliferation, apoptosis, and exocytosis.^1,2^ Moreover, the monitoring of Ca^2+^ concentrations is of significance in organotransplantation, plaque fluid, water quality, soils, and fertilizers.^3^ Light-addressable potentiometric sensors (LAPS) have been reported to detect Ca^2+^ by measuring the potential shift in illuminated areas,^4,5^ offering the possibility of sensing with spatial resolution that could solve the problem of limited active sites in ion-sensitive field-effect transistors (ISFETs)^6^ and overcome the geometry limitation in microelectrode arrays (MEAs).^7^ LAPS are passive devices as they use electrolyte–insulator–semiconductor structures that do not allow a faradaic current to pass. Instead, they measure AC photocurrents that are strongly affected by the charge of ion-selective films placed on the semiconductor surface.

In contrast to LAPS, photoelectrochemical imaging using metal oxide semiconductor substrates such as ITO or α-Fe_2_O_3_ (hematite) is based on the measurement of local light-induced faradaic currents due to the oxidation of hydroxide ions in the solution.^8,9^ Hematite thin films have been shown to be robust sensor substrates for photoelectrochemical imaging of living cells due to their high stability and good spatial resolution.^9^ Hematite is an n-type semiconductor with a bandgap of 1.9–2.2 eV;^10,11^ it is one of the most stable metal oxides under ambient conditions. Hematite has been extensively studied for a wide range of applications, including sensors,^12^ photocatalysts,^13^ lithium batteries,^14^ photoelectrochemical water splitting,^11^ and environmental remediation,^15^ due to their varied shape-dependent properties^16,17^ and the intrinsic properties of α-Fe_2_O_3_, such as nontoxicity, low cost, ease of synthesis, and high resistance to corrosion.^18^ It is anticipated that ion-sensing capability could be developed by surface modification. However, the planar hematite thin film of 200 nm thickness used previously may not be able to produce sufficient photocurrents for ion sensing if coated with a polymeric ion-selective membrane. That is because hematite intrinsically suffers from a short charge carrier lifetime,^11^ a mismatch between the short hole diffusion length (2–4 nm) and the long photon penetration length (∼120 nm at λ = 550 nm),^10^ and a relatively low absorption coefficient (order of 10^3^ cm^–1^), requiring at least a 400–500 nm thick film for optimal light absorption.^18^ Nanostructuring of the hematite planar film to aligned nanorods (NRs) has been reported to improve the photocurrent response as the charge carriers are channelized, facilitating hole transport to the interface, which minimizes the recombination of charge carriers.^19,20^ Moreover, the substrate with a nanostructured rough surface is believed to enhance film adhesion due to additional mechanical interlocking.

**Scheme 1:**
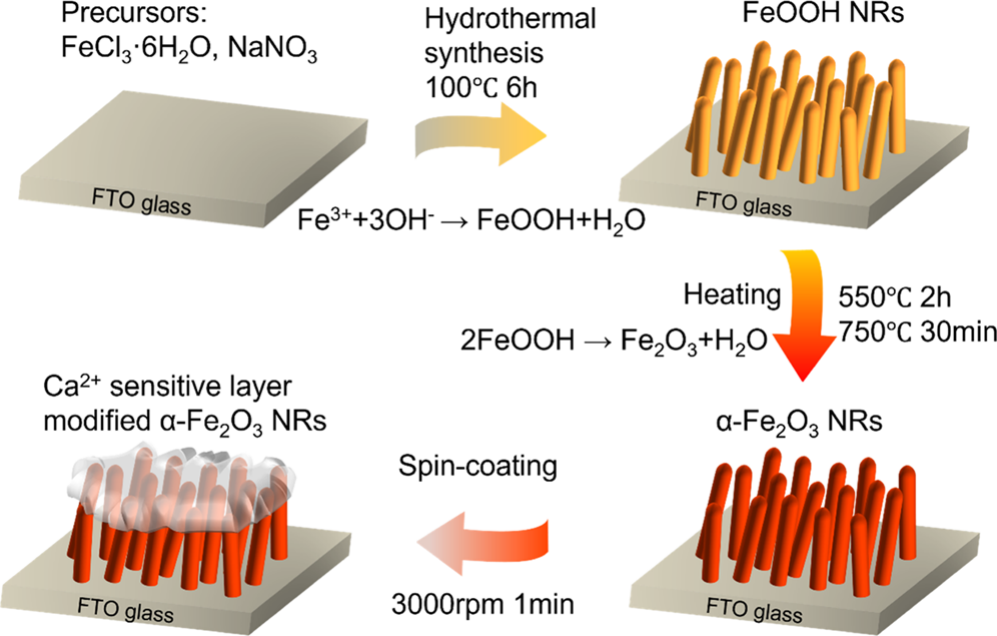
Sensor Preparation Procedure

In this work, hematite NRs synthesized via a simple hydrothermal method (Scheme 1) are proposed as a substrate for photoelectrochemical ion sensing. The bare NRs showed high pH sensitivity, and the polymeric membrane-modified NRs exhibited an amperometric response to Ca^2+^ with a different mechanism to the LAPS device. A high spatial resolution of the hematite NRs was determined by imaging a polymer dot, which reveals the potential of using this system to study ion-channel activities in cell culture in the future.

## Experimental Section

### Materials

Fluorine-doped tin oxide (FTO) glass (15 Ω sq^–1^) was purchased from Solaronix SA, Switzerland. The chemicals for synthesizing hematite NRs include iron(III) chloride hexahydrate (FeCl_3_·6H_2_O, ACS reagent, 97%), sodium nitrate (NaNO_3_, ≥99.0%), and concentrated hydrochloride acid (HCl, ACS reagent, 37%). The chemicals for the preparation of a polymeric membrane include poly(vinyl chloride) (PVC, high molecular weight), dibutyl phthalate (DBP, 99%), dibenzo-18-crown-6 (DB18C6, 98%), and tetrahydrofuran (THF, anhydrous, ≥99.0%). All chemicals were purchased from Sigma-Aldrich. All solutions were prepared using ultrapure water (18.2 MΩ·cm) from a Milli-Q water purification system (Millipore).

### Sample Preparation

The sensor fabrication is illustrated in Scheme 1. Hematite nanorods were synthesized on FTO via an adjusted hydrothermal method.^21,22^ Briefly, FTO glass was cut into 1 cm × 1 cm pieces, and the pieces were subsequently cleaned for 10 min each with acetone, ethanol, and ultrapure water in an ultrasonication bath. After blowing dry with nitrogen gas, the FTO substrate was transferred to a Teflon-lined stainless steel autoclave (50 mL capacity) with 20 mL of an aqueous solution of FeCl_3_·6H_2_O (0.1 M) and NaNO_3_ (1 M) at pH 2 (set by HCl). The autoclave was sealed and maintained at 100 °C for 6 h. After the autoclave cooled down to room temperature naturally, the FeOOH film was rinsed with a copious amount of water and blown dry with nitrogen. Finally, the as-prepared FeOOH film was calcinated at 550 °C for 2 h and 750 °C for 20 min to give crystalline α-Fe_2_O_3_ nanorods. For comparison, hematite planar thin film samples were synthesized based on our previous work.^9^ Poly(methyl methacrylate) (PMMA, average M.W. 120 000) was dissolved in methoxybenzene to form a 20 wt % solution. A PMMA dot was drop coated on hematite NRs and naturally dried before measurement.

The ion-sensitive membrane was prepared following a previously reported procedure:^23,24^ 120 mg of poly(vinyl chloride) (PVC), 10 mg of dibutyl phthalate (DBP), and 10 mg of dibenzo-18-crown-6 (DB18C6) were dissolved in 5 mL of tetrahydrofuran (THF). The mixture was spin-coated on the hematite NRs at 3000 rpm for 1 min. The resulting samples were conditioned in 0.1 M CaCl_2_ solution for 48 h before the test.

### Material Characterization

The surface and cross-sectional morphology of hematite nanorods were characterized using a scanning electron microscope (SEM, FEI Inspect F). Transmission electron microscopy (TEM) images and selected area electron diffraction were obtained by a JEOL-2010 TEM with an acceleration voltage of 200 kV. Ultraviolet–visible (UV–vis) spectra were obtained using a UV–vis spectrometer (PerkinElmer, Lamda 950). X-ray photoelectron spectroscopy (XPS) was carried out by the Nexsa XPS system (Thermo Scientific, U.K.); XPS data were collected and analyzed by Avantage (Thermo Scientific) software. The X-ray diffraction (XRD) analysis was carried out using a PANalytical X’Pert Pro diffractometer configured for grazing incidence X-ray diffraction (GIXRD) with Cu Kα1 radiation. The water contact angle measurement was conducted using a Drop Shape Analysis System (Krüss DSA100, Germany). Topographic imaging was carried out using an atomic force microscope (AFM, Bruker Dimension Icon, U.K.). Mott–Schottky plots and impedance spectra were recorded in Dulbecco’s phosphate-buffered saline (DPBS) solution (pH 7.4) with an Autolab PGSTAT30/FRA2 (Windsor Scientific Ltd., U.K.) using a platinum electrode and an Ag/AgCl electrode as the counter and reference electrodes, respectively. A sinusoidal modulation of 10 mV in amplitude was used at frequencies from 0.1 Hz to 10 kHz.

### Linear Sweep Voltammetry

Chopped light linear sweep voltammetry (LSV) was carried out in DPBS solution (pH 7.4) using an Autolab PGSTAT30/FRA2 with the same three-electrode system as used for impedance measurements. A diode laser (λ = 405 nm, max 50 mW) chopped in 10 s intervals was used as a light source while recording the LSV curves, and the scan rate was 5 mV s^–1^.

### Photoelectrochemical Sensing and Imaging

The experimental setup for photocurrent measurements (Figure S1, Supporting Information) has been described elsewhere.^9^ In brief, a diode laser (BioRay Coherent, λ = 405 nm, max. power = 50 mW) was used for photocurrent excitation. After being collimated by a custom-made spatial filter, the laser beam was manipulated by an analogue mirror (Mirrorcle Technologies, Inc.) and was focused using an objective lens with a correction ring (Nikon, numerical aperture 0.6) to scan the back surface of the sample for imaging. Photocurrents were measured with an MFLI lock-in amplifier (Zurich Instruments) with a platinum electrode and an Ag/AgCl (3 M KCl) electrode acting as the counter and reference electrodes, respectively. Optical images were taken with a digital CMOS camera (ORCA-Flash4.0 LT, Hamamatsu Photonics Ltd., U.K.). The system was controlled and photocurrents were recorded using a custom-designed program written in LABVIEW.

## Results and Discussion

### Hematite Nanorod Characterization

**Figure 1:**
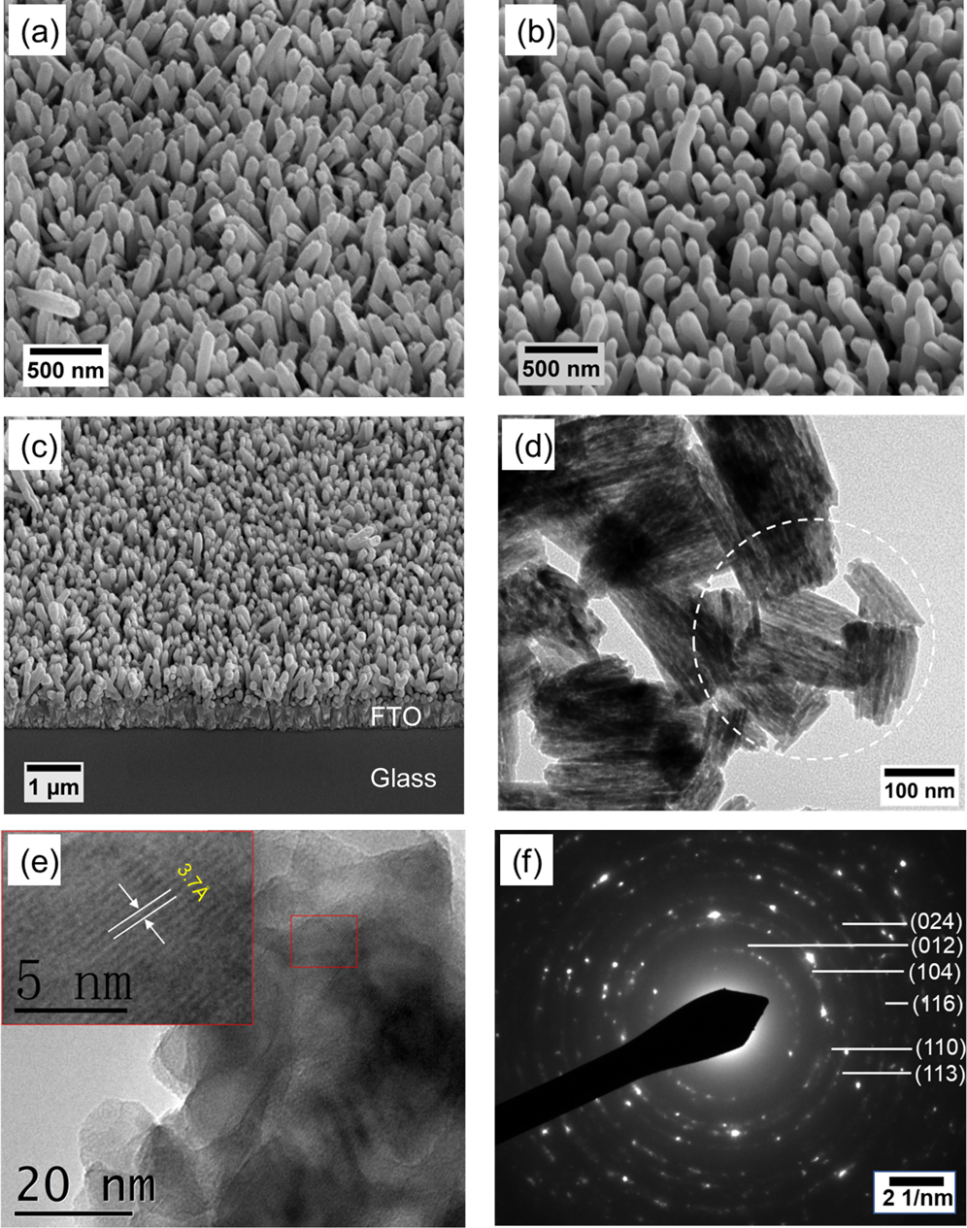
Top-view SEM images of (a) as-prepared FeOOH and (b) annealed α-Fe_2_O_3_ nanorods. (c) Cross-sectional view of α-Fe_2_O_3_ nanorods on the FTO substrate. (d) Bright-field TEM image of α-Fe_2_O_3_ nanorods. (e) High-magnification TEM image of α-Fe_2_O_3_ nanorods; inset: a high-resolution TEM (HRTEM) image taken from the as-obtained a-Fe_2_O_3_ nanorods. (f) Selected area electron diffraction (SAED) pattern of α-Fe_2_O_3_ nanorods.

The morphology of hematite nanorod films was characterized by SEM and TEM. SEM images of the as-prepared FeOOH and annealed α-Fe_2_O_3_ are shown in Figure 1a,b, respectively. Hematite nanorods were arranged in uniform arrays, which were aligned perpendicularly to the FTO substrate. The thickness of the layer was 503.6 ± 35.7 nm, estimated from the cross-sectional SEM image (Figure 1c). This morphology could offer direct electrical pathways for photogenerated carriers and effectively boost electron–hole separation, thus improving the photoelectrochemical properties.^25,26^ The grains in the NRs are smoother compared to those in ref (21) because the samples in this work were heated at higher temperatures; thus, an improved crystallinity and increased size were due to aggregation. The hematite NRs showed a larger size and greater length compared to those in ref (22), which was caused by a different selection and higher concentration of the precursor. A bright-field TEM image of hematite nanorods is shown in Figure 1d, and its corresponding (marked with a white dotted circle in Figure 1d) selected area electron diffraction (SAED) pattern (Figure 1f) reveals the highly polycrystalline nature of α-Fe_2_O_3_ nanorods with observable diffraction rings corresponding to crystallographic planes of hematite (JCPDS 33-0664). Figure 1e shows a high-magnification TEM image of α-Fe_2_O_3_ nanorods and a representative high-resolution TEM (HRTEM) image taken from the hematite nanorods. The lattice fringes are observed with a spacing of ∼3.7 Å, which agrees well with the (012) lattice spacing of hematite.^27^

**Figure 2:**
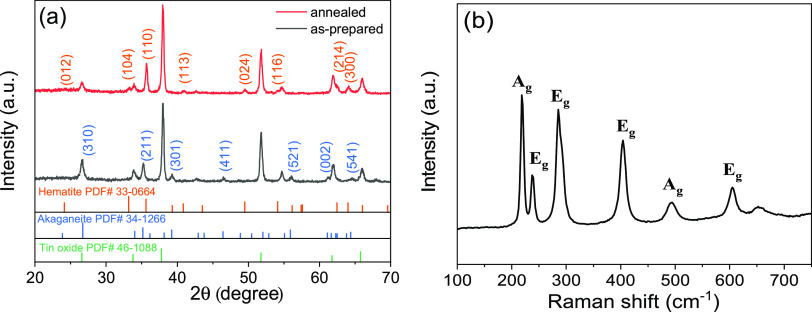
(a) XRD pattern of as-prepared β-FeOOH (akaganeite) and annealed α-Fe_2_O_3_ (hematite) on the FTO substrate. (b) Raman spectrum of hematite NRs on the FTO substrate.

XRD analysis (Figure 2a) confirms that the as-prepared nanorods were β-FeOOH (akaganeite, JCPDS 34-1266), which were then converted into α-Fe_2_O_3_ (hematite, JCPDS 33-0664) via annealing. The strongest (110) diffraction peak indicates that these hematite nanorods have a preferred [110] direction vertical to the substrate, which implies that they were grown along the [110] axis. Hematite with the [110] orientation has been reported to have an anisotropic conductivity that is four orders of magnitude higher and better facilitates charge collection of photo-excited charge carriers along the one-dimensional (1D) nanostructures.^28−30^ The Raman spectrum of hematite NRs (Figure 2b) displays well-established hematite bands (2A_g_ + 5E_g_) located at 222, 244, 289, 406, 497, 605 cm^–1^.^31^ An additional peak at 662 cm^–1^ is possibly due to the presence of nanocrystals.^32,33^ The absence of Raman peaks for β-FeOOH further demonstrates the complete conversion of β-FeOOH into α-Fe_2_O_3_ after calcination.^34^

The surface chemistry of the hematite NRs was revealed by XPS, which confirms the presence of Fe^3+^, O^2–^, and Sn^4+^ in the lattice and hydroxyl groups (−OH) at the surface, and the absence of iron in the XPS spectra of the Ca^2+^-sensitive layer-modified hematite NRs indicates complete coverage of the NRs with the polymer layer (Figure S2). A water contact angle of 34 ± 2° confirmed the hydrophilic character of the NR surface (Figure S3). The topography of the hematite NRs was characterized by AFM (Figure S4), showing a root-mean-square (RMS) roughness of ∼74.2 nm. The optical absorption of hematite NRs was characterized using UV–Vis spectroscopy (Figure S5). A direct bandgap of 2.1 eV was obtained, confirming the feasibility of photocurrent excitation using 405 nm laser illumination. A Mott–Schottky plot revealed a donor density of 6.95 × 10^17^ cm^–3^ and a flat band potential of 0.11 V vs Ag/AgCl for hematite NRs (Figure S6). The charge transfer resistance for hematite NRs determined with electrochemical impedance spectroscopy (EIS) was about 5.6 times lower compared to that of hematite thin films demonstrating that charge transfer at the interface is more effective for NRs (Figure S7).

### Photocurrent Performance of Hematite Nanorods

**Figure 3:**
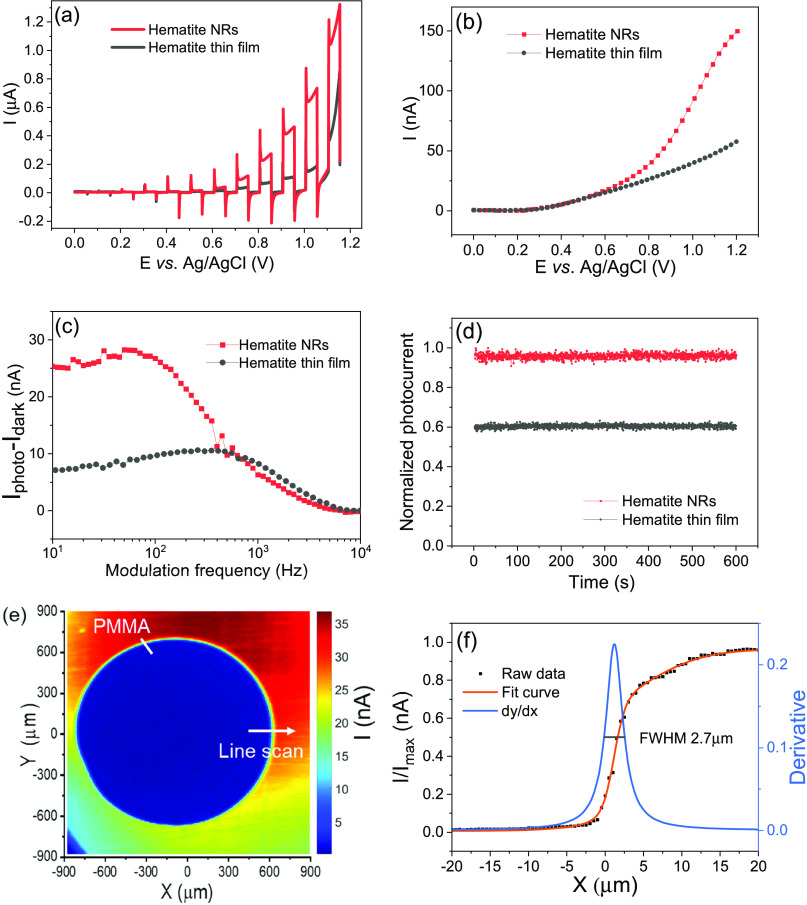
Comparison of photocurrent responses between hematite NRs and hematite planar thin films: (a) LSV curves measured in pH 7.4 DPBS solution with an autolab potentiostat. (b) *I*–*V* curves measured with a focused laser beam modulated at 10 Hz in pH 7.4 DPBS using the PEIS setup. (c) Photocurrent–frequency curves measured with a focused laser beam in pH 7.4 DPBS. (d) *I*–*t* curves measured with a focused laser beam over 600 s. (e) Photocurrent image of a PMMA dot measured at 1.0 V and 1 kHz with a focused laser beam. (f) *X*-axis line scan across the edge of the PMMA dot and a lateral resolution of 2.7 μm was determined from the full width at half maximum (FWHM) of the first derivative of the line scan.

The photocurrent performance of hematite NRs is illustrated and compared with that of a planar hematite thin film reported previously^9^ in Figure 3. Figure 3a shows the chopped light LSV in pH 7.4 DPBS. Hematite NRs and thin films exhibited similar currents at potentials lower than 0.6 V vs Ag/AgCl. However, a significantly higher photocurrent was observed for the NRs at potentials above 0.6 V. The enhanced photoactivity was associated with the high conductivity along the [110] axis and the increased number of active sites due to the NR structure^28,35^ and the high catalytic performance of the hematite (110) facet.^27^ The photocurrent–voltage (*I*–*V*) curves measured with a focused laser beam modulated at 10 Hz in pH 7.4 DPBS using the PEIS setup are shown in Figure 3b. Higher photocurrent values were obtained for the NRs above 0.6 V, reaching 150 nA at 1.2 V compared to 58 nA for the thin film. Figure 3c shows the current–frequency curves measured at 1 V with a focused laser beam in pH 7.4 DPBS. NRs yielded considerably higher net current until the frequency increased to 500 Hz, while at frequencies higher than 500 Hz, there is no significant difference in net photocurrent. Although both samples can be used for imaging at 10–1000 Hz, hematite NRs are the material of choice for pursuing higher photocurrent at low frequencies. Figure 3d depicts the photocurrent–time (*I*–*t*) curves measured at 0.8 V with a focused laser beam modulated at 10 Hz in pH 7.4 DPBS. Both samples were stable over 10 min, indicating good reliability in photoelectrochemical imaging.

### Photocurrent Imaging Using Hematite NRs

Figure 3e shows the photocurrent image of a PMMA dot deposited onto the surface of hematite NRs measured at a modulation frequency of 1 kHz with a bias of 1.0 V. The polymer dot was clearly visible in the photocurrent image showing lower photocurrents compared to the blank surface area owing to the high impedance of the polymer. For lateral resolution measurement, a photocurrent line scan across the edge of the polymer film was conducted with a focused laser beam (Figure 3f). A resolution of 2.7 μm was obtained from the full width at half maximum (FWHM) of the first derivative of the line scan,^36^ which is comparable to the resolution of ITO (2.3 μm)^37^ irradiated with a 405 nm laser and is better than the resolution of InGaN (7 μm).^38^

### Photoelectrochemical Sensing Using Hematite NRs

#### pH Sensitivity of Hematite NRs

**Figure 4:**
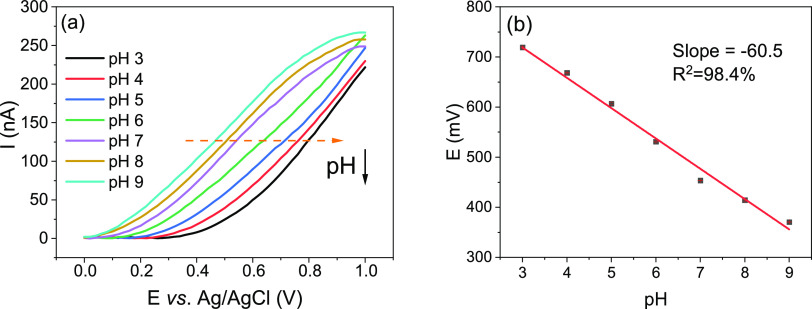
(a) *I*–*V* curves of hematite NRs measured in different pH buffer solutions. (b) Linear fitting shows the pH sensitivity of hematite NRs.

To investigate the pH sensitivity of hematite NRs, photocurrent–voltage (*I*–*V)* curves were recorded at a frequency of 10 Hz in a series of phosphate-buffer solutions (pH 3–9) supplemented with 0.1 M KCl using the PEIS setup. Figure 4a shows that the photocurrent increased with increasing pH in the pH range of 3–9, which reflects the enhanced oxidation reaction of hydroxide ions at higher pH.^37^ A mixed mechanism of ion exchange in a surface layer with hydroxyl (−OH) groups and redox reactions was previously suggested for semiconducting oxides.^39,40^ For hematite NRs, a linear relationship between the applied voltage and pH was observed with a high sensitivity of 60.5 mV/pH (Figure 4b), which could be ascribed to a large number of active sites of the hematite NR structure, indicating the great potential of high-resolution pH imaging using hematite NRs for biochemical applications.

#### Calcium-Ion Sensitivity of Hematite NRs Coated with an Ion-Selective PVC Coating

**Figure 5:**
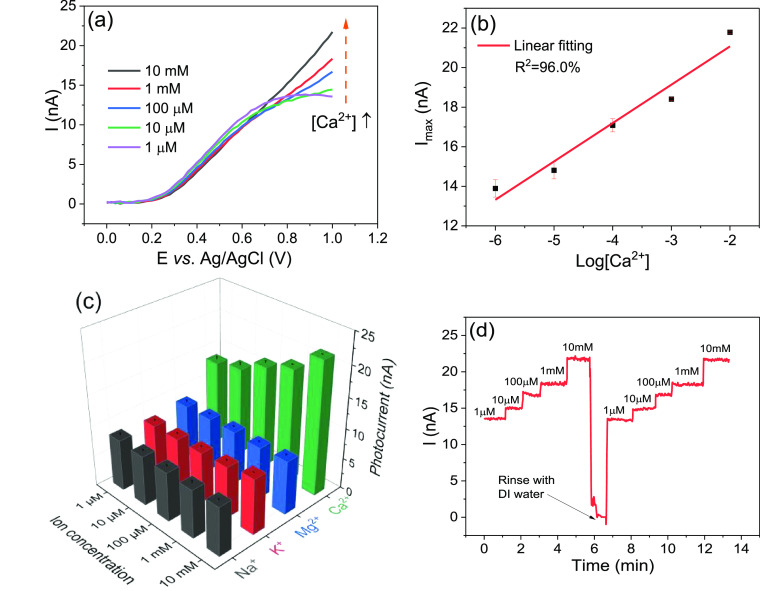
(a) *I*–*V* curves of hematite NRs measured at different Ca^2+^ concentrations and (b) the corresponding calibration curve. (c) Photocurrent responses of the hematite NR-based Ca^2+^ sensor toward different ions. (d) Time trace of the photocurrent responses in Ca^2+^-containing solutions with different concentrations measured at a potential of 1 V.

**Figure 6:**
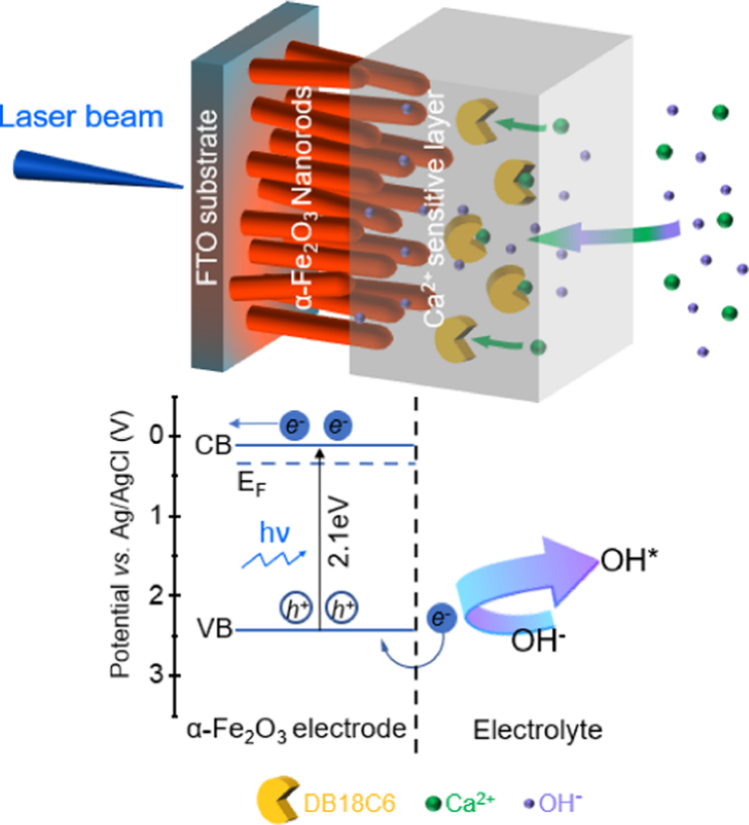
Schematic diagram of the modified hematite NRs for the photoelectrochemical sensing of Ca^2+^.

The morphology of Ca^2+^-sensitive layer-modified hematite NRs is shown in Figure S8. Instead of a thick layer, a thin continuous polymer film was formed following the contour of NRs and filling the gaps between them, which provides the opportunity for ion sensing with faradaic electrochemistry with sensor coatings previously only employed in potentiometric sensors. To explore the Ca^2+^ sensing properties of the sensor, *I*–*V* curves were recorded at different concentrations of CaCl_2_ (0.1 M KCl as supporting electrolyte) with pH around 6.02 ± 0.03 at a modulation frequency of 1 kHz. At potentials lower than 0.7 V, the order of the *I*–*V* curves is in agreement with a potentiometric response. However, this response was relatively small (12.5 mV decade^–1^) and was not reproducible from one device to another. At potentials higher than 0.9 V, the photocurrent increased with the calcium ion concentration from 1 μM to 10 mM (Figure 5a). Figure 5b shows the calibration curve of this amperometric response plotted using an average photocurrent for each concentration at a potential of 1 V. A limit of detection (LOD) of 0.42 μM was obtained from the concentration corresponding to the intersection of the linear fit of the calibration data and a line through data points in a concentration range where the sensor shows no response to calcium ions (Figure S9).^41^ The diameter of solvated calcium ions (7 Å) is too large to be accommodated in the cavity of DB18C6 (4 Å), but the high charge on the oxygen atoms of DB18C6 allows the complexation of Ca^2+^ with DB18C6, known as the ion–dipole interaction;^42−44^ thus, a PVC film with DB18C6 has previously shown good selectivity to calcium ions in ion-selective electrodes.^23,24,45^ While the uncoated hematite device clearly showed a mixed potentiometric and amperometric response mechanism to pH, for the PVC-coated sensor, a potentiometric response could be expected as the insulating properties of PVC can block faradaic currents making this into a LAPS device. However, this only applies to low potentials. At higher potentials, a pure amperometric response was observed for the first time with these types of sensor coatings. As more calcium ions bind to DB18C6, the concentration of counter ions, including hydroxide ions, in the film increases, thus enhancing the oxidation reaction of hydroxide ions at the hematite surface (see the schematic diagram in Figure 6). The selectivity of the hematite NR-based Ca^2+^ sensor was examined against magnesium (Mg^2+^), potassium (K^+^, 0.1 M NaCl as supporting electrolyte), and sodium (Na^+^) ions with the same concentration as Ca^2+^ (Figure 5c). Significantly higher photocurrents were observed with Ca^2+^ owing to the high binding affinity of Ca^2+^ with DB18C6, and there is no observable response toward Mg^2+^, K^+^, and Na^+^. As DB18C6 is not expected to bind these ions, their concentration in the film is likely to be significantly smaller than that of Ca^2+^, resulting in a negligible sensor response. Good reversibility was shown in the time trace of photocurrent responses in Ca^2+^-containing solutions with different concentrations (Figure 5d). Photocurrents increase with the Ca^2+^ concentration and can return to their original levels after several washes of the electrolyte cell with deionized water. Figure S10 shows *I*–*V* curves of three sensors, each measured three times in 1 mM CaCl_2_ solution. All of the measurements showed an almost identical response indicating good reliability and stability due to the good stability of the hematite substrate and the rough surface of NRs enhancing the adhesion of the PVC membrane. This is an advantage over PVC-coated silicon sensors, which show poor adhesion between the PVC membrane and silicon.^46,47^ A control experiment was conducted by measuring a bare hematite NR sample (Figure S11a); no response to Ca^2+^ was found, confirming that the Ca^2+^ sensitivity was derived from the coating. The Ca^2+^-sensitive PVC membrane was coated on a hematite thin film for comparison; small photocurrent values and high noise level make it unsuitable for sensor application (Figure S11b).

## Conclusions

Hematite NRs aligned vertically to the FTO substrate fabricated by a hydrothermal method were studied as a platform for photoelectrochemical imaging and sensing. Hematite NRs showed a significantly increased (about 2.6 times at 1.2 V) photocurrent under irradiation of a focused 405 nm laser compared to a hematite planar film with good stability. The photocurrent imaging of a polymer dot with a high spatial resolution of 2.7 μm was achieved. Hematite NRs displayed a pH sensitivity of 60.5 mV/pH over the pH range of 3–9 without surface modification, indicating a mixed potentiometric and amperometric response mechanism. When modified with a thin Ca^2+^-sensitive PVC membrane, hematite NRs showed an amperometric response toward Ca^2+^ rather than the potentiometric response measured with thicker films on traditional LAPS devices and displayed good selectivity over Na^+^, K^+^, and Mg^2+^. The ion-selective film showed significantly improved adhesion on the rough hematite NR surface compared to traditional silicon-based LAPS substrates. In the future, the Ca^2+^ sensitivity of the hematite NR sensor, in conjunction with its imaging capabilities, could be used to study ion-channel activities in cell culture through real-time photocurrent imaging. It is envisaged that a multifunctional/multiplexed platform that can detect various analytes spatiotemporally can be achieved by further modification. The successful utilization of nanostructured semiconductors for photoelectrochemical imaging and sensing tremendously expands the range of photoresponsive nanomaterials that could be explored in this field.

## Supporting Information Available

Schematic of the photoelectrochemical imaging system setup; XPS spectra of hematite NRs and the Ca^2+^-sensitive layer-modified hematite NRs; atomic concentrations of different chemical species from the fit of XPS spectra; water contact angle of hematite NRs; AFM images of hematite NRs; UV–vis spectrum of hematite NRs and the Tauc plot; Mott–Schottky plot of hematite NRs; electrochemical impedance spectra of hematite NRs and the equivalent circuit; SEM image of Ca^2+^-sensitive layer-modified hematite NRs; calibration curve of a Ca^2+^ sensor to determine the lower limit of detection; *I*–*V* curves of three sensors, each measured three times in 1 mM CaCl_2_ solution; and control experiments of bare hematite NRs and the Ca^2+^-sensitive layer-modified hematite planar film in Ca^2+^ sensing (PDF)
