# Supplementary material for: Photoelectrochemical Detection of Calcium Ions Based on Hematite Nanorod Sensors
Source: ACS Appl Nano Mater. 2022 Nov 10;5(11):17087–94. doi: 10.1021/acsanm.2c03978 (PMC9706496; doi:10.1021/acsanm.2c03978)
Supplement: Supplementary file 1 — an2c03978_si_001.pdf [file an2c03978_si_001.pdf]

Supporting information

Photoelectrochemical detection of calcium ions based on hematite nanorod sensors

Bo Zhou[1], Yunlu Jiang[2], Qian Guo[1], Anirban Das[1], Ana Belén Jorge Sobrido[1], Karin A. Hing[1], Anatoly V. Zayats[2], Steffi Krause[1,*]

[1]School of Engineering and Materials Science, Queen Mary University of London, Mile End Road, London E1 4NS, UK

[2]Department of Physics and London Centre for Nanotechnology, King's College London, Strand, London WC2R 2LS, UK

s.krause@qmul.ac.uk

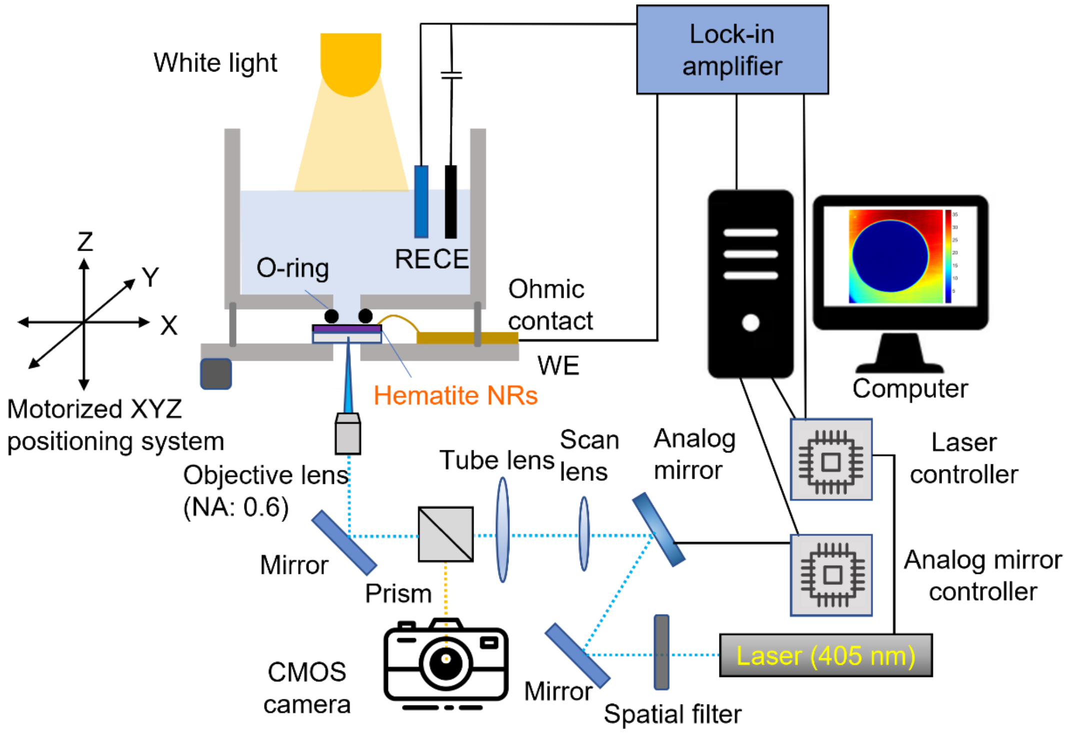

Figure S1. Schematic of the photoelectrochemical imaging system (PEIS) setup.

The surface electronic structure and chemical states of hematite NRs and Ca^{2+} sensitive layer modified-hematite NRs were investigated by XPS measurements. The survey spectra (Figure S2a) confirm the presence of Fe, O, Sn and adventitious C at the surface of hematite NRs and the presence of C, O Cl at the modified surface. The elemental compositions of these two surfaces are summarised in Table S1. The high-resolution spectrum of Fe2p of hematite NRs (Figure S2b) contains two distinct peaks at binding energies of ~ 711.1 eV for Fe 2p3/2 and ~ 724.4 eV for Fe 2p1/2, and two shake-up satellites at ~ 719.3 eV and ~ 733.4 eV were observed as indicators of Fe^{3+} in Fe_2O_3 [1]. However, there is no detectable peak at this range for the modified hematite NRs which confirms the full coverage of the polymer layer (Figure S2b). O1s spectrum of the hematite NRs (Figure S3c) displays an intense peak associated with the "O^{2-}" of the crystalline network at 530.1 eV and a peak located at 531.9 eV which is attributed to hydroxyl groups at the surface with atomic ratio 83.5% to 16.5% [2]. While there is a main O1s peak for the modified surface associated with the organic C-O bond (86.8%) appears in dibenzo-18-crown-6 (DB18C6) and dibutyl phthalate (DBP), and a small peak related to the organic C=O bond (13.2%) of DBP (Figure S3c). Peaks with spin-orbit splitting (Δ=1.6 eV) in Cl2p spectrum (Figure S2d) of the modified surface confirms the assignment of organic Cl from polyvinyl chloride (PVC).

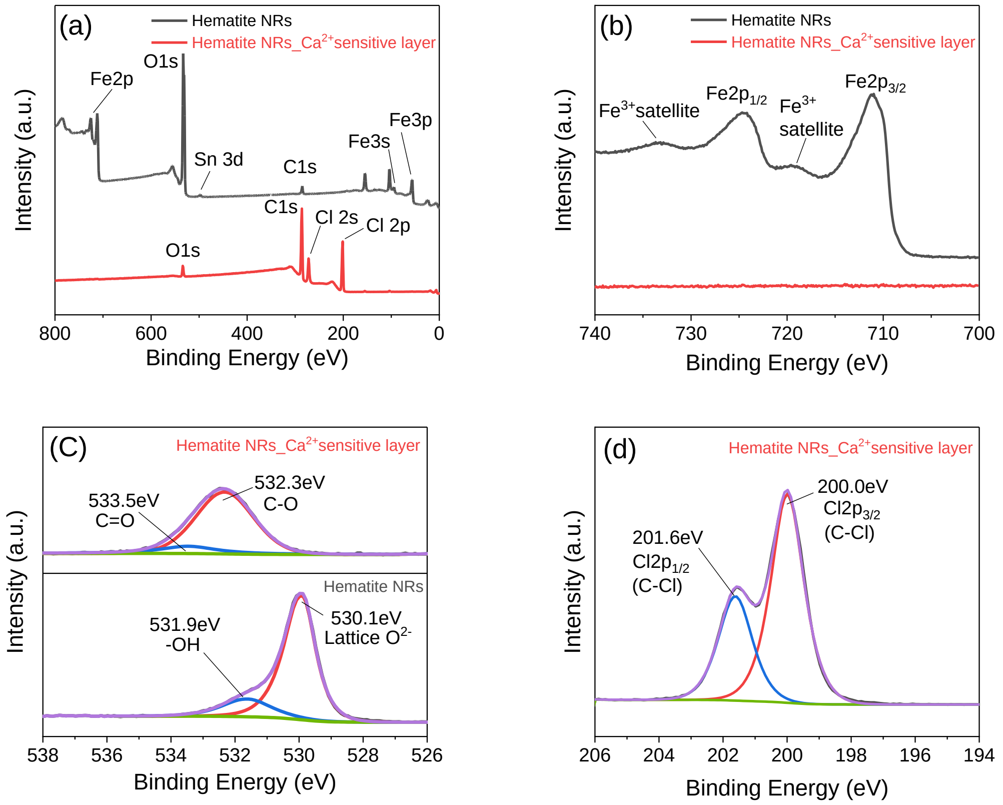

Figure S2. XPS spectra of hematite NRs and Ca^{2+} sensitive layer modified-hematite NRs:(a) survey scans, (b) high-resolution Fe2p scans, (c) high-resolution O1s scans, (d) high-resolution Cl2p scan.

Table S1. Atomic concentrations of different chemical species from fit of XPS spectra.

Sample / Atomic concentration / Peak	Hematite NRs_Ca^{2+} sensitive layer		Hematite NRs	
C1s	73.5%		7.5%	
O1s	4.7%	C-O: 86.8%	75.1%	O^{2-}: 83.5%
		C=O: 13.2%		-OH: 16.5%
Cl2p	18.8%		0%	
Fe2p	0%		17.2%	
Sn3d	0%		0.2%	

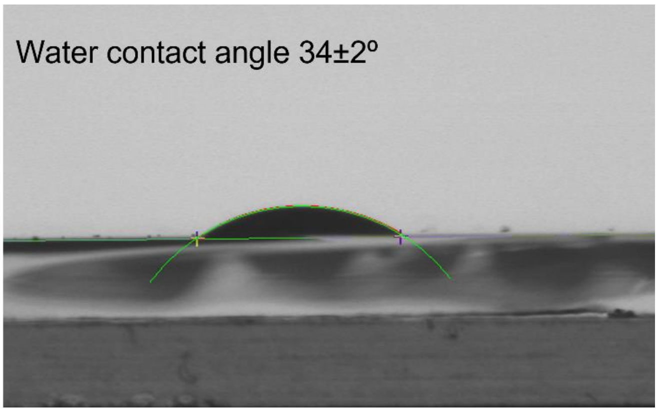

Figure S3. Water contact angle of hematite NRs.

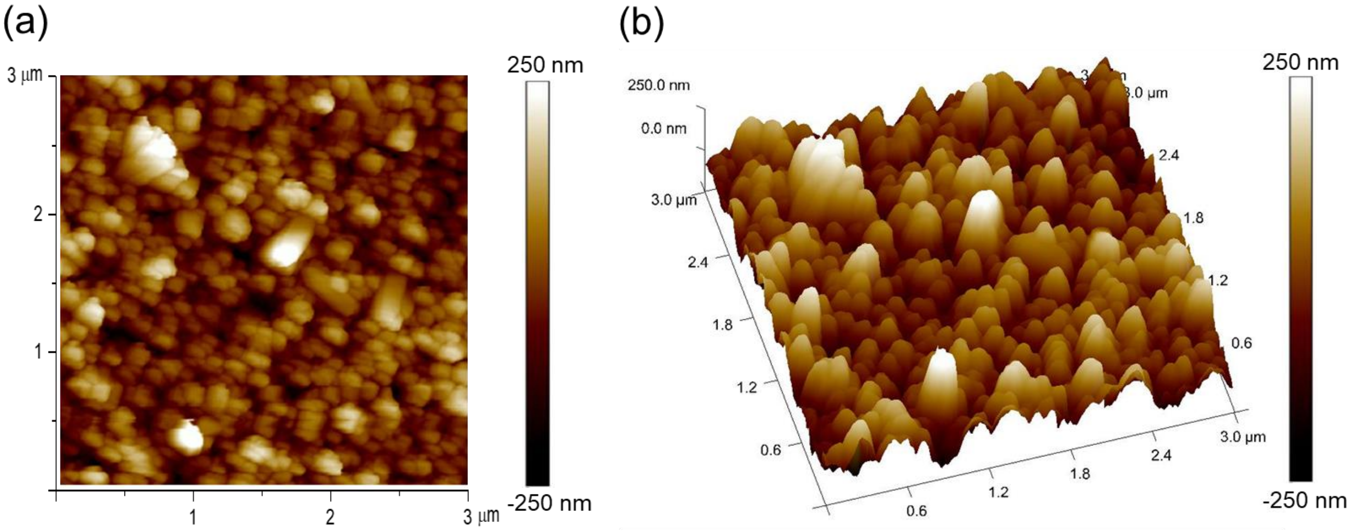

Figure S4. (a) AFM top view image and (b) 3D map of hematite NRs.

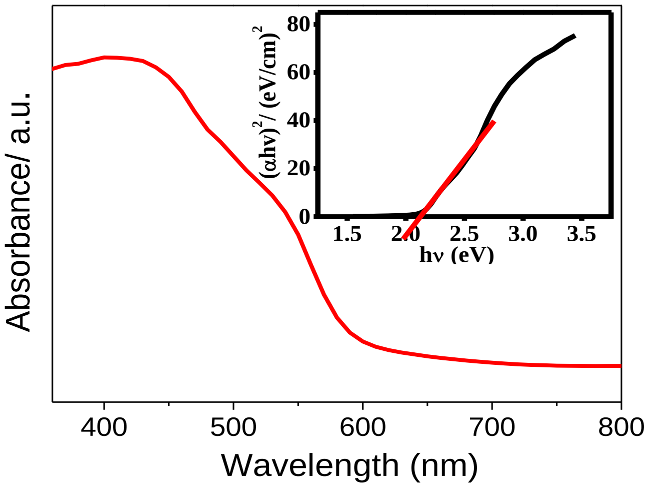

Figure S5. UV-vis spectrum of α-Fe_2O_3 nanorods and inset Tauc-plot.

From a Mott−Schottky plot of a hematite film (Figure S6), a donor density of 6.95 × 10^{17} cm^{-3} and flat band potential (U_{FB}) of 0.11 V vs. Ag/AgCl were calculated based on the Mott–Schottky equation (equation S1):

$$\frac{1}{c^2} = \frac{1}{e\varepsilon\varepsilon_0 N_d}\left(U - U_{FB} - \frac{KT}{e}\right) \qquad \text{(S1)}$$

where C is the space charge layer capacitance, e is the electron charge, ε is the relative permittivity of hematite (ε = 80) [3], ε_0 is the permittivity of vacuum, N_d is the donor density, U_{FB} is the flat band potential, U is the applied potential at the electrode, K is the Boltzmann's constant, and T is the operation temperature.

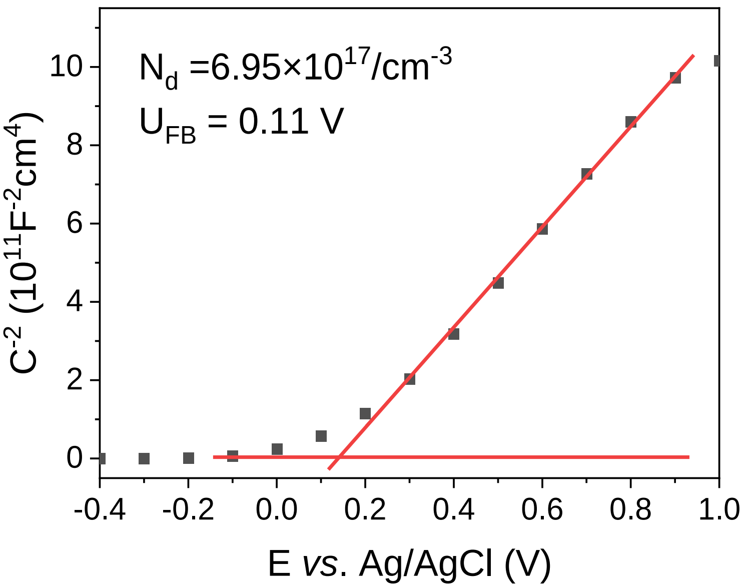

Figure S6. Mott−Schottky plot of hematite NRs recorded at 1 kHz using an Autolab PGSTAT30/FRA2 with a sinusoidal voltage perturbation of 10 mV in pH 7.4 DPBS.

EIS measurements were conducted to investigate the kinetics of charge transfer at the electrode surface. Figure S7a shows the Bode plots of a hematite nanorod sample and a hematite thin film sample measured at 0.8 V under illumination. Hematite NRs exhibit lower impedance than thin film sample at low frequencies, which indicates NRs have higher charge transfer efficiency. Figure S7b shows the equivalent circuit model with two time constants for fitting the EIS data, which includes the electrolyte resistance R_s, a resistance representing the trapping of holes in surface states R_{trap}, and a charge transfer resistance $R_{ct,ss}$, and two constant phase element (CPE) Q_{sc} and Q_{ss} associated with space charge capacitance and surface state capacitance respectively [4]. The values of frequency power n for the CPE are closer to 1 (perfect capacitor) for hematite thin films compared than those values for NRs, which reflects the inhomogeneous distribution of reaction rate or higher surface roughness due to the NR structure [5, 6]. The calculated values of the circuit elements are presented in Table S1; a higher Q_{ss} for NRs compared to thin film indicates more charging of the surface states with hematite NRs, and about 5.6 times lower $R_{ct,ss}$ for hematite NRs represents a more effective charge transfer from surface states to the electrolyte.

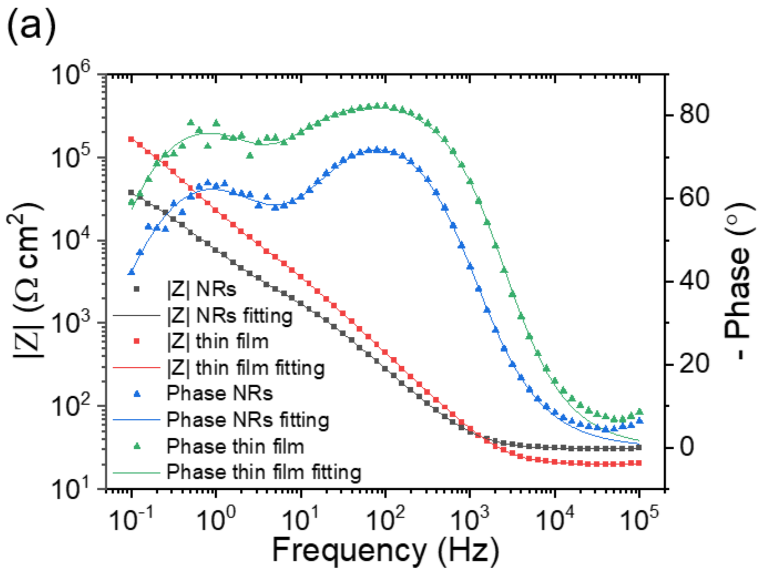

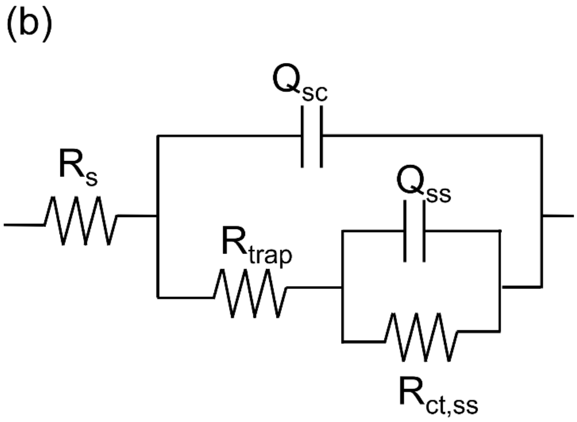

Figure S7. (a) Bode plots of hematite NR and thin film electrodes measured at 0.8 V in the dark and under illumination in pH 7.4 DPBS (dots - measured values, lines - equivalent circuit fit); (b) Equivalent circuit model used for fitting the impedance spectra.

Table S2. Parameters calculated from the equivalent circuit.

	R_s (Ω cm^2)	Q_{sc} (S Sec^n/cm^2)	R_{trap} (Ω cm^2)	Q_{ss} (S Sec^n/cm^2)	$R_{ct,ss}$ (Ω cm^2)
Hematite NRs	30.43	9.99×10^{-6} n=0.91	3.41×10^{3}	2.07×10^{-5} n=0.82	6.27×10^{4}
Hematite thin film	19.87	4.96×10^{-6} n=0.95	1.82×10^{4}	3.26×10^{-5} n=0.92	3.51×10^{5}

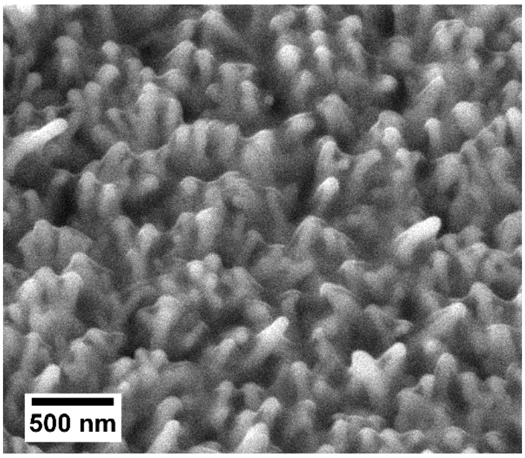

Figure S8. SEM image of Ca^{2+} sensitive layer modified hematite NRs.

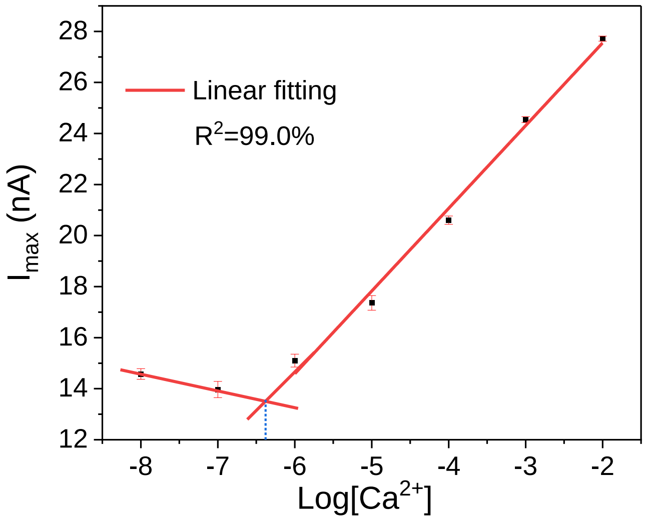

Figure S9. A calibration curve of a Ca^{2+} sensor to determine the lower limit of detection. The results were obtained with a sensor from a different batch fabricated under the same conditions as the sensors used for the data presented in Figure 5.

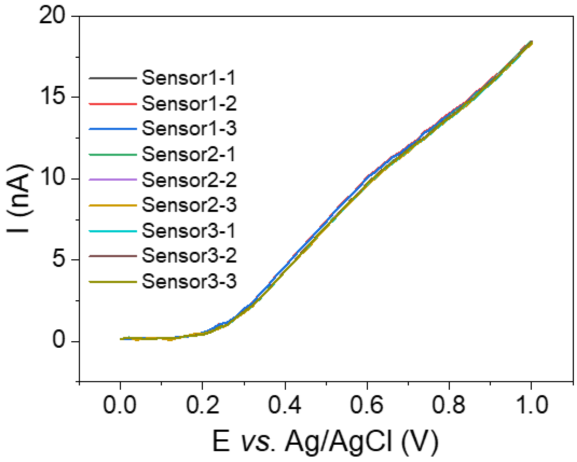

Figure S10. I-V curves of three sensors each measured three times in 1 mM $CaCl_2$ solution.

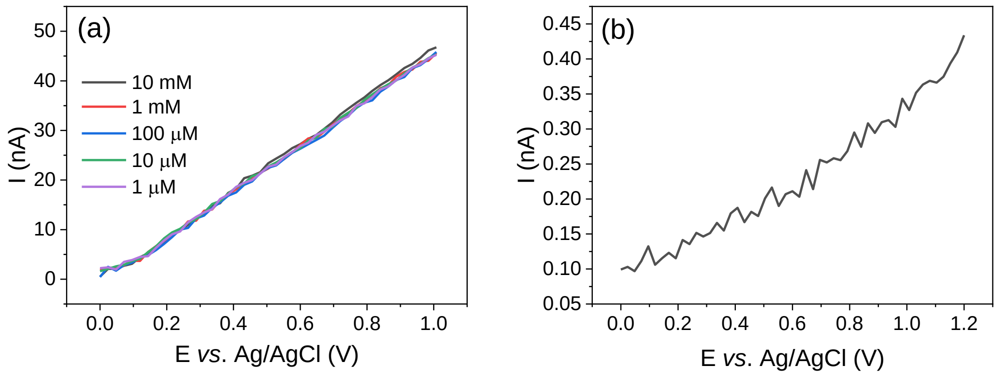

Figure S11. (a) I-V curves of a bare hematite NRs measured in $CaCl_2$ solution. (b) I-V curve of a PVC-coated hematite thin film measured in 10 mM $CaCl_2$ solution.

References

1. Biesinger, M. C.; Payne, B. P.; Grosvenor, A. P.; Lau, L. W. M.; Gerson, A. R.; Smart, R. S. C., Resolving surface chemical states in XPS analysis of first row transition metals, oxides and hydroxides: Cr, Mn, Fe, Co and Ni. *Appl. Surf. Sci.* **2011,** *257* (7), 2717-2730.
2. Dupin, J.-C.; Gonbeau, D.; Vinatier, P.; Levasseur, A., Systematic XPS studies of metal oxides, hydroxides and peroxides. *PCCP* **2000,** *2* (6), 1319-1324.
3. Hankin, A.; Bedoya-Lora, F. E.; Alexander, J. C.; Regoutz, A.; Kelsall, G. H., Flat band potential determination: avoiding the pitfalls. *Journal of Materials Chemistry A* **2019,** *7* (45), 26162-26176.
4. Klahr, B.; Gimenez, S.; Fabregat-Santiago, F.; Hamann, T.; Bisquert, J., Water oxidation at hematite photoelectrodes: the role of surface states. *J. Am. Chem. Soc.* **2012,** *134* (9), 4294-302.
5. Kim, C.-H.; Pyun, S.-I.; Kim, J.-H., An investigation of the capacitance dispersion on the fractal carbon electrode with edge and basal orientations. *Electrochim. Acta* **2003,** *48* (23), 3455-3463.
6. Mulder, W. H.; Sluyters, J. H.; Pajkossy, T.; Nyikos, L., Tafel current at fractal electrodes: Connection with admittance spectra. *Journal of Electroanalytical Chemistry and Interfacial Electrochemistry* **1990,** *285* (1), 103-115.
